# Thrombus Imaging Characteristics to Predict Early Recanalization in Anterior Circulation Large Vessel Occlusion Stroke

**DOI:** 10.3390/jcdd11040107

**Published:** 2024-03-29

**Authors:** Nerea Arrarte Terreros, Jeffrey Stolp, Agnetha A. E. Bruggeman, Isabella S. J. Swijnenburg, Ricardo R. Lopes, Laura C. C. van Meenen, Adrien E. D. Groot, Manon Kappelhof, Jonathan M. Coutinho, Yvo B. W. E. M. Roos, Bart J. Emmer, Ludo F. M. Beenen, Diederik W. J. Dippel, Wim H. van Zwam, Ed van Bavel, Henk A. Marquering, Charles B. L. M. Majoie

**Affiliations:** 1 Department of Biomedical Engineering and Physics, Amsterdam University Medical Centers, Location University of Amsterdam, Meibergdreef 9, 1105 AZ Amsterdam, The Netherlandse.vanbavel@amsterdamumc.nl (E.v.B.);; 2 Department of Radiology and Nuclear Medicine, Amsterdam University Medical Centers, Location University of Amsterdam, 1105 AZ Amsterdam, The Netherlands; 3 Department of Neurology, Amsterdam University Medical Centers, Location University of Amsterdam, 1105 AZ Amsterdam, The Netherlands; j.stolp@amsterdamumc.nl (J.S.);; 4 Department of Neurology, Erasmus Medical Center, 3015 GD Rotterdam, The Netherlands; 5 Department of Radiology and Nuclear Medicine, Maastricht University Medical Center, 6229 HX Maastricht, The Netherlands; w.van.zwam@mumc.nl

**Keywords:** early recanalization, transferred patients, intravenous thrombolysis, endovascular treatment, acute ischemic stroke

## Abstract

The early management of transferred patients with a large vessel occlusion (LVO) stroke could be improved by identifying patients who are likely to recanalize early. We aim to predict early recanalization based on patient clinical and thrombus imaging characteristics. We included 81 transferred anterior-circulation LVO patients with an early recanalization, defined as the resolution of the LVO or the migration to a distal location not reachable with endovascular treatment upon repeated radiological imaging. We compared their clinical and imaging characteristics with all (322) transferred patients with a persistent LVO in the MR CLEAN Registry. We measured distance from carotid terminus to thrombus (DT), thrombus length, density, and perviousness on baseline CT images. We built logistic regression models to predict early recanalization. We validated the predictive ability by computing the median area-under-the-curve (AUC) of the receiver operating characteristics curve for 100 5-fold cross-validations. The administration of intravenous thrombolysis (IVT), longer transfer times, more distal occlusions, and shorter, pervious, less dense thrombi were characteristic of early recanalization. After backward elimination, IVT administration, DT and thrombus density remained in the multivariable model, with an AUC of 0.77 (IQR 0.72–0.83). Baseline thrombus imaging characteristics are valuable in predicting early recanalization and can potentially be used to optimize repeated imaging workflow.

## 1. Introduction

Current treatment options for acute ischemic stroke (AIS) patients with large vessel occlusions (LVOs) are intravenous thrombolysis (IVT) and endovascular treatment (EVT) [1,2]. IVT can induce rapid recanalization prior to EVT, may increase the rate of successful reperfusion with EVT, and can dissolve thrombi located in regions not reachable by EVT [2,3,4]. However, the administration of IVT before EVT increases the risk of hemorrhages and potentially increases the chances of thrombus fragmentation [5,6]. In addition, IVT has been proven to be less effective in proximal large vessel occlusions (LVOs) compared to more distal occlusions [3], while EVT can successfully be targeted at proximal LVOs, especially those that are refractory to IVT [7].

In LVO-AIS patients transferred from primary stroke centers (PSCs), early recanalization (i.e., dissolution of the LVO on neuroimaging at the CSC, relinquishing the need for EVT) following IVT is reported to occur in 20–40% of all cases [8,9,10]. This underlines the importance of IVT in transferred LVO-AIS patients. Being able to predict the PSC at which patients will or will not recanalize early might improve treatment planification, leading to optimized resource expenditure and cost reduction [11,12]. In the case of the accurate prediction of early recanalization, futile inter-hospital transfers (and therefore capacity constraints at the CSC) could be limited. Determining which patients will not recanalize early can help with speeding up the workflow to the angio-suite room, by, e.g., avoiding the need to perform repeated imaging at CSC admission, which, on average, takes 20 min longer [13]. 

Thrombus characteristics, such as size or composition, play a major role in the recanalization process [14,15,16,17,18,19], and therefore, can be determinant of treatment effect and patient outcome. Such characteristics can be assessed on baseline radiological imaging. An early-stage assessment of these characteristics may guide treatment selection. The goal of our study is to identify predictors of early recanalization in transferred AIS patients based on baseline patient clinical and thrombus imaging characteristics.

## 2. Materials and Methods

### 2.1. Study Design and Patient Selection 

We included patients with definite LVO at PSC who had early recanalization on repeated imaging at CSC. Our database consists of adult (≥18 years) stroke patients, who were referred to our CSC (Amsterdam University Medical Centers, University of Amsterdam, Amsterdam, The Netherlands) for EVT between January 2016 and January 2021. We excluded late-window patients who presented at the CSC later than 6 h after symptom onset.

The patients were part of our regional prospective stroke database, in which all EVT referrals from the 11 PSCs in our region (catchment area ~3.4 million) were collected. Early recanalization was defined as the resolution of the proximal LVO (internal carotid artery (ICA); middle cerebral artery (MCA) M1 and proximal M2 segments; anterior cerebral artery (ACA) A1 and proximal A2 segments) or its migration to a distal location not eligible for EVT (distal M2, M3, distal A2, A3). 

The reasons to undergo repeated imaging mainly include clinical improvement or deterioration [13]. Therefore, non-early recanalized patients from the repeated imaging group are suspected to be non-representative of commonly transferred LVO patients. Since baseline imaging data of patients transferred to our center without repeated imaging were not available, consecutive non-early recanalized transferred patients from the MR CLEAN Registry were collected for comparison. The MR CLEAN Registry is a multicenter prospective observational registry of all patients undergoing EVT for AIS in The Netherlands. Our hospital is part of this registry. 

From the MR CLEAN Registry, we selected adult (≥18 years) stroke patients with an LVO of the anterior circulation that were referred to a CSC for EVT between March 2014 and November 2017, were presented at the CSC within 6 h after symptom onset and had baseline thrombus imaging measurements available. These patients had a persistent LVO, as confirmed by the first digital subtraction angiography run (for the transferred patients in the Registry, repeated imaging was not available). 

### 2.2. Ethical Approval

For the regional database, this study was evaluated by the medical ethics review committee of the Amsterdam University Medical Centers, location AMC, who waived the need for obtaining written informed consent. The procedures followed were all in accordance with institutional guidelines. A letter with detailed information about the study was sent to all patients meeting the inclusion criteria. The patient or legal representative had the opportunity to deny consent for use of their data via an opt-out form, conforming to the European Union General Data Protection Regulation. 

The MR CLEAN Registry was approved by the central medical ethics committee of the Erasmus Medical Center Rotterdam, which served as the review board of all participating centers and granted permission to carry out the study as a registry (MEC-2014–235). All patients or legal representatives were provided with oral and written information on the registry and had the opportunity to withdraw consent to use their data. 

### 2.3. Patient and Thrombus Imaging Characteristics

#### 2.3.1. Regional Database

Clinical and imaging data of patients from between January 2016 and June 2019 were already available [8]. For patients included between June 2019 and January 2021, additional measurements and data collection were performed following the same procedures as stated previously [8].

The National Institute of Health Stroke Scale (NIHSS) was used to determine stroke severity at baseline. If not reported by the treating physician, the NIHSS was scored retrospectively from written neurological examinations, in accordance to previously published methods [20].

The workflow metrics collected were time from stroke onset to arrival at the PSC, time from stroke onset to imaging at the PSC, time from arrival to the PSC to initiation of IVT, time from arrival at the PSC to arrival at the CSC, and time from IVT initiation to arrival at the CSC.

Thrombus imaging characteristics included occlusion location, the distance from the terminus of intracranial carotid artery to the thrombus (DT), thrombus length, thrombus density, and thrombus perviousness. 

**Figure 1:**
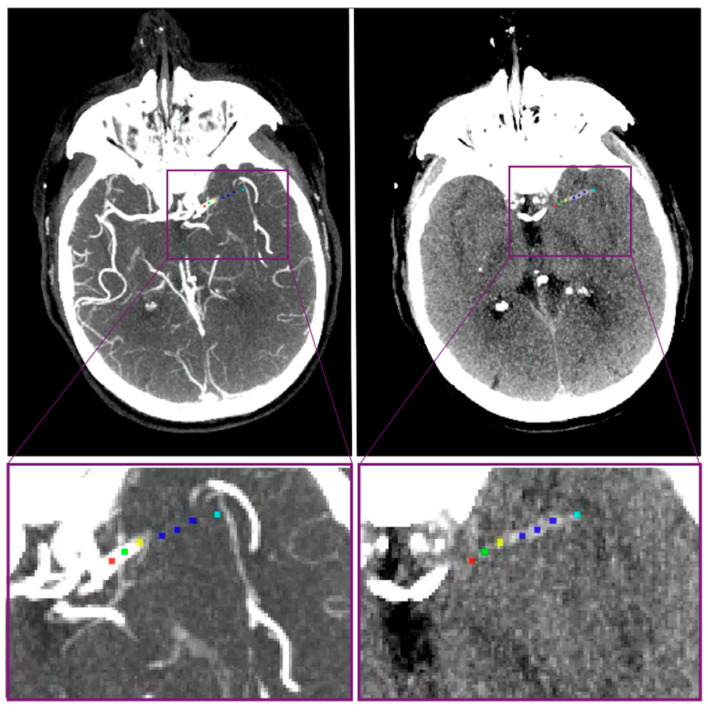
Thrombus imaging characteristics. Manually placed markers to compute thrombus characteristics. **Left**: thick-slab maximum intensity projection of a CT angiography showing a sudden stop of contrast due to the occlusion. **Right**: thick-slab maximum intensity projection of a non-contrast CT where the hyperdense artery sign of the thrombus is visible. Red marker: placed at internal carotid artery terminus (ICA-T). Green markers: placed between ICA-T and proximal border of the thrombus. Yellow marker: proximal thrombus border. Blue markers: proximal, mid, and distal parts of the thrombus. Cyan marker: distal thrombus border.

The location of the intracranial LVO was assessed on CTA and subsequently compared with imaging from the PSC by experienced neuroradiologists from the CSC during routine clinical work-up. All included patients were eligible for EVT on baseline CT imaging with an occlusion of the ICA, the M1 vessel segment, the proximal M2 segment of the MCA or A1 segment, and the proximal A2 segment of the ACA. Also, the presence of an ipsilateral extracranial internal carotid artery stenosis/occlusion was scored as part of the routine clinical work-up. The method to measure DT, length, density, and perviousness has previously been described [8]. Baseline non-contrast CT and CT angiography data were coregistered using Elastix software [21]. Measurements were performed on ITK snap software, which permits on to simultaneously visualize and place markers on both CT scans [22]. Markers were manually placed along the occluded vessel centerline at the following locations: ICA terminus, proximal and distal thrombus borders, and proximal, mid, and distal parts of the thrombus (Figure 1). In case of curved and tortuous vessels, additional markers were placed between the ICA terminus and the proximal thrombus border. These measurements were performed by 3 trained observers: NAT, ISJS, and AAEB.

With these markers, DT and thrombus length were acquired by computing the Euclidean distance between adjacent markers. Thrombus density and perviousness were computed as the average intensities over a 1 mm-radius region of interest at the proximal, mid, and distal thrombus markers. Thrombus perviousness is a proxy of permeability and assesses how much contrast penetrates the thrombus. 

If coregistration errors were present, DT and thrombus length were measured by placing markers in one of the CT scans and using the other one as a reference. Thrombus perviousness and density were only measured in good-quality thin-slice CT data (i.e., images without movement, metal and beam hardening artefacts, incomplete field of view, excessive noise, and poor contrast opacification). 

The modified Rankin scale score at 90 days, with a score ranging from 0 to 6 (0 being no symptoms and 6 being death) was also registered to assess differences in functional outcome. Good functional outcome was defined as an mRS between 0 and 2. A symptomatic intracerebral hemorrhage (sICH) was defined as hemorrhage resulting in neurological deterioration with an increase in NIHSS ≥ 4 [23].

#### 2.3.2. MR CLEAN Database

The data collection and thrombus imaging characteristics methods from the MR CLEAN Registry have already been described in detail previously [24,25]. The followed patient workup is in line with the one described here (our hospital is one of the MR CLEAN centers). 

### 2.4. Statistical Analysis 

Our analysis consists of (1) comparing early-recanalized LVO (ER-LVO) and non-early-recanalized LVO (NER-LVO) patients and (2) building a logistic regression model to predict early recanalization using clinical and imaging data available at the PSC. To perform the statistical analysis in the group comparison, we used R (version 4.2.0). To build the prediction model, we used SciPy and Scikit-learn libraries from Python (version 3.6). 

#### 2.4.1. Group Comparison

We compared baseline patient clinical and thrombus imaging characteristics between ER-LVO and NER-LVO patients. Numerical data are presented as median and interquartile range (IQR) and categorical data as number and proportion (%). The Mann–Whitney U test was used to compare numerical data, and the $\chi^{2}$ and Fisher’s exact tests for categorical data. Post-hoc analyses were adjusted with Bonferroni corrections for multiple testing. Statistical significance was set at *p* < 0.05. 

#### 2.4.2. Prediction Model

We built a multivariable binary logistic regression model to predict early recanalization using both clinical and thrombus imaging variables available at the PSC. We initially included all variables for which the difference had a *p* < 0.1 in the group comparison. The collinearity of these characteristics was assessed by computing Spearman’s pairwise correlation coefficients (ρ) for two numerical (or categorical ordinal) variables, the Cramer’s V coefficient for two categorical variables, and Point–biserial correlation coefficients (r_pb_) for a binary and a numerical variable. Strongly correlated variables (|ρ| > 0.6, V > 0.6, |r_pb_| > 0.6) were removed from the model. In addition, parameters were excluded from the model using manual backward elimination. 

To fairly assess the predictive value of our model, we built the model on training sets and assessed the model’s performance on testing sets. To minimize potential biases due to the data splitting, we validated the predictive ability of the models by computing the median of the area under the curve (AUC) of the receiver operating characteristics (ROC) curve for 100 5-fold cross-validations; i.e., 100 times, we split the database into 5 groups, and took each group as a testing set (20% of all data), while the remaining groups were used as a training set (80% of all data). Calibration curves (or reliability diagrams) were built using the whole dataset to compare the predicted and true probabilities of early recanalization. We additionally reported the sensitivity (true ER-LVO rate) and specificity (true NER-LVO rate) of the model. Patients with missing data were excluded.

## 3. Results

### 3.1. Patient and Thrombus Imaging Characteristics 

We included 81 ER patients and 322 patients from the registry with a persistent LVO. (Supplemental Figure S1). In 43/81 patients, the thrombus completely dissolved, and in 38/81 patients the thrombus migrated to a more distal segment of the vasculature, too distal to be treated with EVT. Spontaneous recanalization (defined as early recanalization without IVT) occurred in 6/81 patients: 3 patients with complete thrombus dissolution, and 3 patients with a thrombus that was too distally migrated for EVT. 

ER-LVO patients showed more often IVT administrations (93% vs. 78%, *p* < 0.01) and had lower NIHSS scores at CSC presentation (5 vs. 16, *p* < 0.01) than NER-LVO patients. The workflow-related time metrics show that in the ER-LVO group, the arrival at PSC to arrival at CSC time (157 min vs. 101 min, *p* < 0.01) and the IVT initiation to arrival at CSC time (126 min vs. 76 min, *p* < 0.01) were longer compared to the NER-LVO group. ER-LVO patients tended to be older (75 years vs. 71 years, *p* = 0.05) and had lower rates of hypertension (35% vs. 46%, *p* = 0.08), but these differences were not significant (Table 1). 

A chart of thrombus imaging measurements in the early-recanalized group can be found in Supplemental Figure S2. For the registry patients, such a chart is presented in [25]. ER-LVO patients had fewer ICA occlusions (1% vs. 23%, *p* < 0.01 after Bonferroni correction) and more M2 occlusions (32% vs. 13%, *p* < 0.01 after Bonferroni correction). This was also reflected in the DT measure: ER-LVO patients had more distal thrombi (DT of 23 mm vs. 10 mm, *p* < 0.01). We also found that ER-LVO patients had shorter (16 mm vs. 18 mm, *p* = 0.02), more pervious (12 HU vs. 5 HU, *p* < 0.01) and less dense thrombi (43 HU vs. 50 HU, *p* < 0.01) compared to NER-LVO patients (Table 1).

We found that ER-LVO patients tend to have better functional outcomes (mRS 0–2, 52% vs. 38%, *p* = 0.06) and fewer occurrences of sICH (0% vs. 8%, *p* = 0.07), but these differences were not significant (Table 1). 

### 3.2. Prediction Model

**Figure 2:**
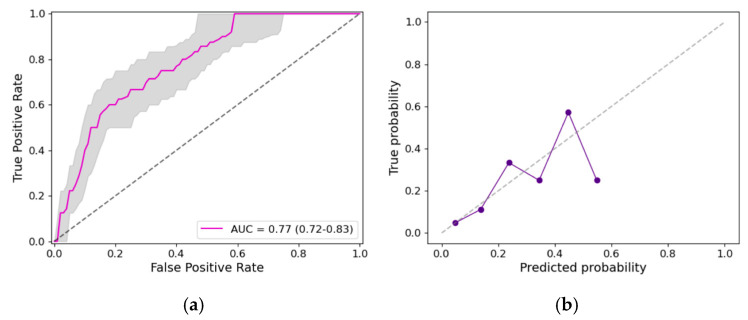
Model performance and calibration curves. (**a**) Median (magenta) and interquartile range (grey) of the ROC curve for 100 5-fold cross-validations. Median AUC was 0.77 (IQR 0.72–0.83). (**b**) Calibration curve showing the true probability over the predicted probability. AUC, area under the curve; ROC, receiver operating characteristics.

We considered the following variables available at PSC admission with a *p* < 0.1 in the group comparison: age, history of hypertension, IVT administration, occlusion location, DT, thrombus length, perviousness, and density. Correlation analysis showed that DT and occlusion location (ρ = 0.66, *p* < 0.01) were strongly correlated (Supplemental Figure S3)—therefore, we did not include occlusion location. The results before and after backward elimination can be found in Table 2. After backward elimination, we found that IVT administration (OR 4.3, 95% CI 1.2–15.4, *p* = 0.03), DT (OR 1.03, 95% CI 1.01–1.05 per mm, *p* < 0.01) and thrombus density (OR 0.94, 95% CI 0.90–0.97 per HU, *p* < 0.01) remained in the model (Table 2). The median (and IQR) ROC curve of the 100 5-fold cross-validations and calibration curves are displayed in Figure 2. The median AUC of the ROC curve was 0.77 (IQR 0.72–0.83). For a specificity of 70%, we achieved a sensitivity of 71%.

## 4. Discussion

In our study, IVT administration, longer door-to-door times, more distal occlusions, and shorter, more pervious, less dense thrombi were associated with early recanalization. Our model for early recanalization prediction based on clinical and thrombus imaging variables collected at PSC admission showed moderate discriminative ability. Assessing the imaging characteristics of the thrombus at an early stage may support clinical evidence and contribute to the optimization of transferred patients’ workflow. 

In an earlier study, we compared thrombus and patient characteristics between early-recanalized and non-early-recanalized LVO-AIS transferred patients that underwent repeated imaging from our regional prospective stroke registry [8]. In our current study, we have shown that thrombus imaging characteristics and IVT administration are significant predictors for early recanalization in transferred patients. 

Our current study also shows an indication that early recanalization is time-dependent: ER-LVO patients had longer times between IVT administration and CSC presentation. This association needs to be further elucidated: rural areas with long inter-hospital transfer times might have higher rates of ER than a highly urbanized setting, like our region.

In respect to other workflow times, we did not show an association between onset-to-IVT time and early recanalization. In other research, a shorter onset-to-IVT time was associated with higher rates of recanalization [26]—thrombi are suspected to grow [27], become stiffer and contract over time, challenging IVT penetration [28]. One possible explanation for our contrasting result is our relatively fast onset-to-IVT times, with medians well below 100 min in both groups. 

A significant number of patients undergo futile inter-hospital transfers, thus incurring extra costs [11], and this may cause unnecessary capacity problems for ambulance services, but also for neurointerventional teams who have to stand ready for every transferred patient. The predicted probabilities of early recanalization were shown to significantly affect the cost-effectiveness of transfers to a CSC [12]. Given the moderate predictive ability of our model at PSC admission, the transportation to a CSC (and therefore, EVT treatment) should not be ruled out for a potential ER-LVO patient. 

The critical variables of the prediction model are expected based on common sense and previous literature. The value of the presented study partly resides in the actual quantification of the ER-LVO characteristics, and the future incorporation of such information in risk models. Such models should account for the information gain versus time loss, and could be used to better plan repeated imaging in transferred patients. Patients with a high likelihood of early recanalization could directly undergo repeated imaging to confirm the prediction. Notably, this is already done in the clinic: in 50% of cases, the reason to undergo repeated imaging is clinical improvement [13]; that is, a decrease in the NIHSS score between PSC and CSC admission. However, not all patients that have clinical improvements recanalize early. A prediction model like the one presented in this study supports this observation by accounting for multiple relevant variables simultaneously, including thrombus imaging characteristics. Determining ER-LVO patients at an early stage can reduce futile transfers to the angio-suite room and the associated invasive imaging and procedural risks. For patients expected in the NER-LVO group, the need for repeated imaging can be minimized, reducing the delay between the CSC door and the angio-suite room by 20 min on average [13]. A recent trial on patients directly presented at the CSC showed that direct to angio-suite workflow led to improved clinical outcomes and decreased costs compared to the conventional imaging workflow [29].

### Limitations 

This study has several limitations. The ER-LVO patients were selected from transferred patients undergoing repeated imaging. The reasons to undergo repeated imaging are not arbitrary and include clinical improvement (52%), clinical deterioration (24%) and other reasons (24%) [13]. Therefore, the non-early recanalized patients from the repeated-imaging database were suspected to not be representative of the commonly transferred patients eligible for EVT. Thus, the MR CLEAN Registry database was used. Our main limitation is that the patients included in this study do not belong to the same study population, although both include stroke patients treated in The Netherlands, and our center is one of the MR CLEAN centers. In addition, the inclusion time periods are different (2014–2017 vs. 2016–2021). 

The NIHSS scores at PSC admission were not available for the NER-LVO group. Due to the lack of thin-slice and good-quality images, a large number of patients had to be excluded for the thrombus imaging measurements, particularly for perviousness and density. 

Stroke etiology, which was not included in our model, might be a relevant variable to consider in future prediction models of early recanalization. 

A substantial amount of mRS scores were missing, especially in the ER-LVO group (38%). The main reason for this was because transferred patients in the ER-LVO group that showed clinical improvement were sent back to the PSC immediately; therefore, the contact information required to get an adequate follow-up was lacking. Additionally, the COVID-19 pandemic might also have affected the collection of ER-LVO follow-up data. The collection of 90-day mRS scores is part of the MR CLEAN Registry protocols, and therefore, fewer patient outcomes were missing (10%).

The predictive ability of our models is moderate, which might be limited by the number of patients included or by our ability to identify or measure the most relevant factors associated with early recanalization. In addition, our logistic regression model did not account for non-linearities in the model’s features, and the use of more advanced prediction models (machine learning) could potentially improve the accuracy. 

Two of the parameters that are included in the prediction model, DT and thrombus density, require manual measurements. Automated measurements could further help in the clinical workflow to make the model more practical to use.

## 5. Conclusions

IVT administration, longer door-to-door times, more distal occlusions, and shorter, more pervious, less dense thrombi are associated with early recanalization. Thrombus imaging characteristics, specifically thrombus distality and density, play a major role in the prediction of early recanalization. These characteristics can be used to predict early recanalization with moderate discriminative ability, and ultimately, to better plan repeated imaging workflow.

## Figures and Tables

**Table 1:** Baseline characteristics, thrombus imaging characteristics and patient functional outcome.

Baseline Clinical Characteristics *	ER-LVO, n = 81	NER-LVO, n = 322	*p*-Value
Age (years)—median (IQR)	**75 (63–85)**	**71 (62–80)**	**0.05**
Sex, female—no./total (%)	45/81 (56)	166/322 (52)	0.52
Medical history—no./total (%)			
Previous stroke	17/80 (21)	54/317 (17)	0.38
Diabetes mellitus	16/80 (20)	54/319 (17)	0.52
Hypertension	28/80 (35)	142/310 (46)	0.08
Atrial fibrillation	14/80 (18)	79/317 (25)	0.16
Pre-stroke mRS 0–2—no./total (%)	59/81 (92)	289/314 (92)	0.97
Systolic blood pressure ^α^ (mmHg)—median (IQR)	149 (130–166)	150 (133–164)	0.61
Diastolic blood pressure ^β^ (mmHg)—median (IQR)	82 (76–90)	80 (70–90)	0.31
NIHSS_CSC_^γ^– median (IQR)	**5 (2–10)**	**16 (12–20)**	**<0.01**
IVT—no./total (%)	**75/81 (93)**	**251/322 (78)**	**<0.01**
**Workflow related timing variables—median (IQR)**	**ER-LVO,** **n = 81**	**NER-LVO, n = 322**	***p*-value**
Onset ^†^-to-PSC-door ^δ^ (min)	50 (35–96)	52 (39–97)	0.52
Onset-to-PSC-imaging ^ε^ (min)	67 (52–118)	67 (47–110)	0.72
PSC door-to-IVT ^ζ^ (min)	25 (18–33)	24 (18–33)	0.79
PSC door to CSC door ^η^ (min)	**157 (122–240)**	**101 (81–128)**	**<0.01**
IVT-to-CSC door ^θ^ (min)	**126 (92–178)**	**76 (56–97)**	**<0.01**
**Thrombus imaging characteristics ^‡^**	**ER-LVO,** **n = 81**	**NER-LVO, n = 322**	***p*-value**
Occlusion location—no./total (%)			**<0.01**
ICA	**1/81 (1)**	**74/320 (23)**	
M1	53/81 (65)	201/320 (63)	
M2	**26/81 (32)**	**43/320 (13)**	
A1/A2	1/81 (1)	2/320 (1)	
DT ^ι^ (mm)—median (IQR)	**23 (14–32)**	**10 (0–21)**	**<0.01**
Thrombus length ^κ^ (mm)—median (IQR)	**16 (11–21)**	**18 (12–30)**	**0.02**
Thrombus perviousness ^λ^ (HU)—median (IQR)	**12 (4–21)**	**5 (−2–12)**	**<0.01**
Thrombus density ^λ^ (HU)—median (IQR)	**43 (36–52)**	**50 (44–56)**	**<0.01**
**Patient functional outcome**	**ER-LVO,** **n = 81**	**NER-LVO, n = 322**	***p*-value**
mRS at 90 days			**<0.01**
0	**8/50 (16)**	**15/293 (5)**	
1	12/50 (24)	40/293 (14)	
2	6/50 (12)	57/293 (19)	
3	3/50 (6)	46/293 (16)	
4	4/50 (8)	33/293 (11)	
5	5/50 (10)	15/293 (5)	
6	12/50 (24)	87/293 (30)	
Good functional outcome (mRS 0–2) at 90 days	26/50 (52)	112/293 (38)	0.06
sICH	0/81 (0)	13/322 (4)	0.07

Missing values: ^α^ 14, ^β^ 15, ^γ^ 22, ^δ^ 127, ^ε^ 52, ^ζ^ 185, ^η^ 126, ^θ^ 190, ^ι^ 10, ^κ^ 16, and ^λ^ 43. * Scored at PSC unless stated otherwise. ^†^ Witnessed stroke onset or, if unknown, time the patient was last seen well. ^‡^ Characteristics scored at baseline CT imaging (at PSC). BL, baseline; CSC, comprehensive stroke center; CT, computed tomography; DT; distance from intracranial carotid artery terminus to the thrombus; HU, Hounsfield units; IQR, interquartile range; IVT, intravenous treatment with alteplase; mm, millimeters; mRS, modified Rankin Scale; NIHSS, National Institute of Health Stroke Scale; PSC, primary stroke center; sICH, symptomatic intracranial hemorrhage.

**Table 2:** Predictive model of early recanalization. Results of the multivariable binary logistic regression model before and after backward elimination.

**Prediction model**	**Before Backward Elimination, n = 348**		
	**Characteristics Associated with Early Recanalization**	**Odds Ratio (95% CI)**	***p*-Value**
	Age (per year)	1.01 (0.98–1.04)	0.29
	Hypertension	0.45 (0.20–1.01)	**0.05**
	IVT administration	3.77 (1.04–13.64)	**0.04**
	DT (per mm)	1.03 (1.01–1.06)	**<0.01**
	Thrombus length (per mm)	1.00 (0.97–1.03)	0.98
	Thrombus perviousness (per HU)	1.01 (0.98–1.04)	0.40
	Thrombus density (per HU)	0.94 (0.90–0.98)	**<0.01**
	**After backward elimination, n = 360**		
	**Characteristics associated with early recanalization**	**Odds ratio (95% CI)**	***p*-value**
	IVT	4.3 (1.2–15.4)	**0.03**
	DT (per mm)	1.03 (1.01–1.05)	**<0.01**
	Thrombus density (per HU)	0.94 (0.90–0.97)	**<0.01**

CI, Confidence Interval; DT, distance from intracranial carotid artery terminus to the thrombus; IVT, intravenous thrombolysis with alteplase; mm, millimeters.

## Data Availability

All data relevant to the study are included in the article or have been uploaded as Supplementary Materials. The data that support the findings of this study are available upon reasonable request, after clearance by the local ethics committee.

## Supplementary Materials

The following supporting information can be downloaded at: https://www.mdpi.com/article/10.3390/jcdd11040107/s1, Figure S1: Inclusion flowcharts; Figure S2: Early recanalization thrombus imaging characteristics inclusion flowchart; Figure S3: Correlation analysis.

## Abbreviations

ACA	anterior cerebral artery
CI	confidence interval
CSC	comprehensive stroke center
CT	computed tomography
CTA	computed tomography angiography
DT	distance from intracranial carotid artery terminus to the thrombus
ER-LVO	early recanalized large vessel occlusion
EVT	endovascular treatment
HU	Hounsfield units
IQR	interquartile range
ICA	internal carotid artery
IVT	intravenous treatment with alteplase
IVT to CSC door	time from intravenous treatment with alteplase initiation to arrival at the comprehensive stroke center
mm	millimeters
MCA	middle cerebral artery
mRS	modified Rankin scale
NCCT	non-contrast computed tomography
NER-LVO	non-early recanalized large vessel occlusion
NIHSS	National Institute of Health Stroke Scale
Onset to PSC door	time from stroke onset to arrival at the primary stroke center
Onset to PSC imaging	time from stroke onset to imaging at the primary stroke center
PSC	primary stroke center
PSC door to CSC door	time from arrival at the primary stroke center to arrival at the comprehensive stroke center
PSC door to IVT	time from arrival at the primary stroke center to initiation of intravenous treatment with alteplase sICH, symptomatic intracranial hemorrhage
