# Supplementary material for: Thrombus Imaging Characteristics to Predict Early Recanalization in Anterior Circulation Large Vessel Occlusion Stroke
Source: J Cardiovasc Dev Dis. 2024 Mar 29;11(4):107. doi: 10.3390/jcdd11040107 (PMC11050543; doi:10.3390/jcdd11040107)
Supplement: Supplementary file 1 [file jcdd-11-00107-s001.zip › jcdd-2927772-supplementary.pdf]

SUPPLEMENTAL MATERIAL

Figures

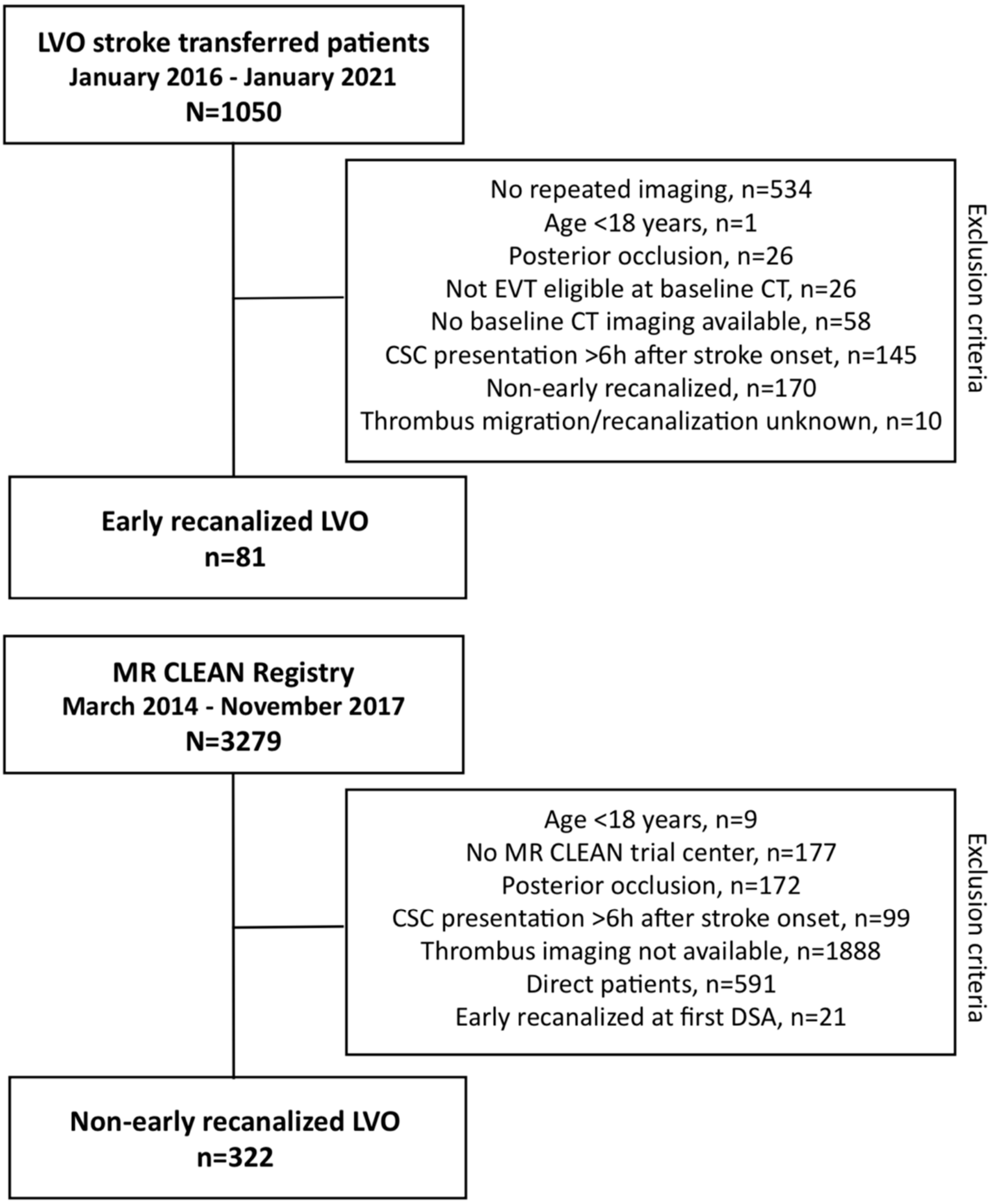

Supplemental Figure S1: Inclusion flowcharts. *CSC, comprehensive stroke center; CT, computed tomography; DSA, digital subtraction angiography; EVT, endovascular treatment; LVO, large vessel occlusion.*

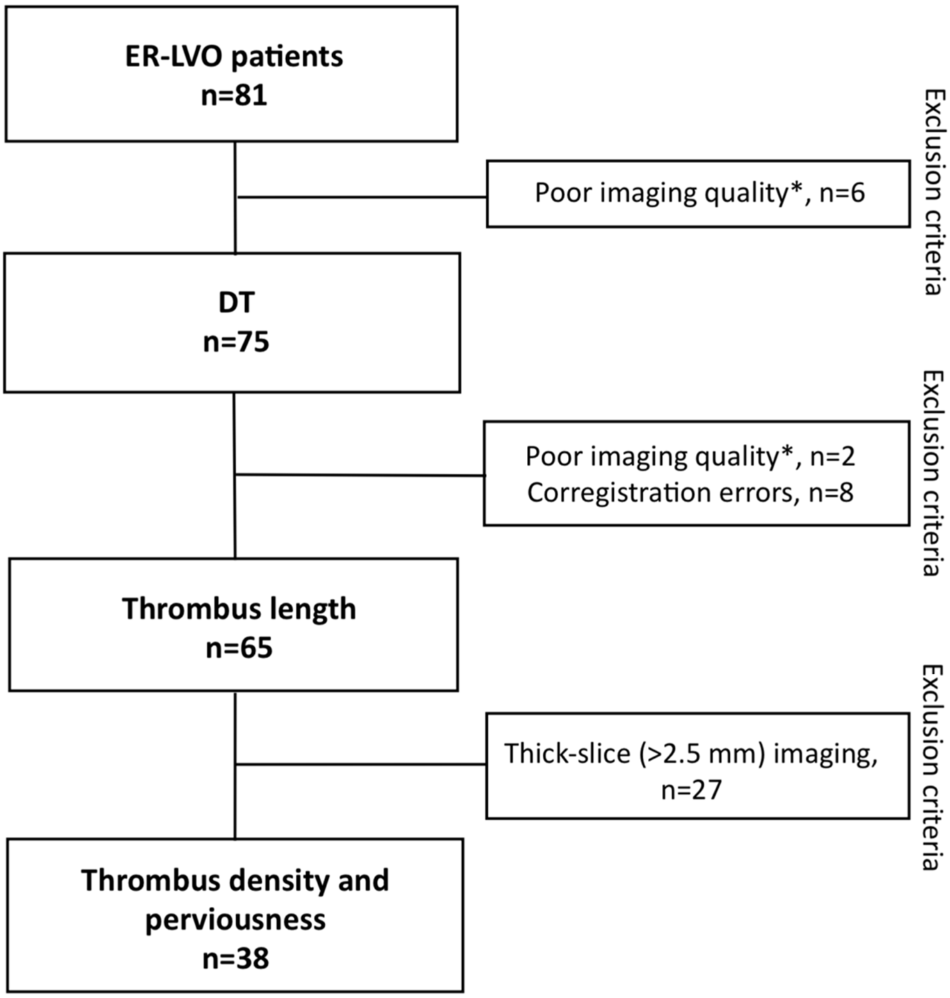

Supplemental Figure S2: Early recanalization thrombus imaging characteristics inclusion flowchart. *movement, metal and beam hardening artefacts, incomplete field of view, excessive noise, and poor contrast opacification in computed tomography angiography scan. *DT, distance from the intracranial carotid artery terminus to the thrombus.*

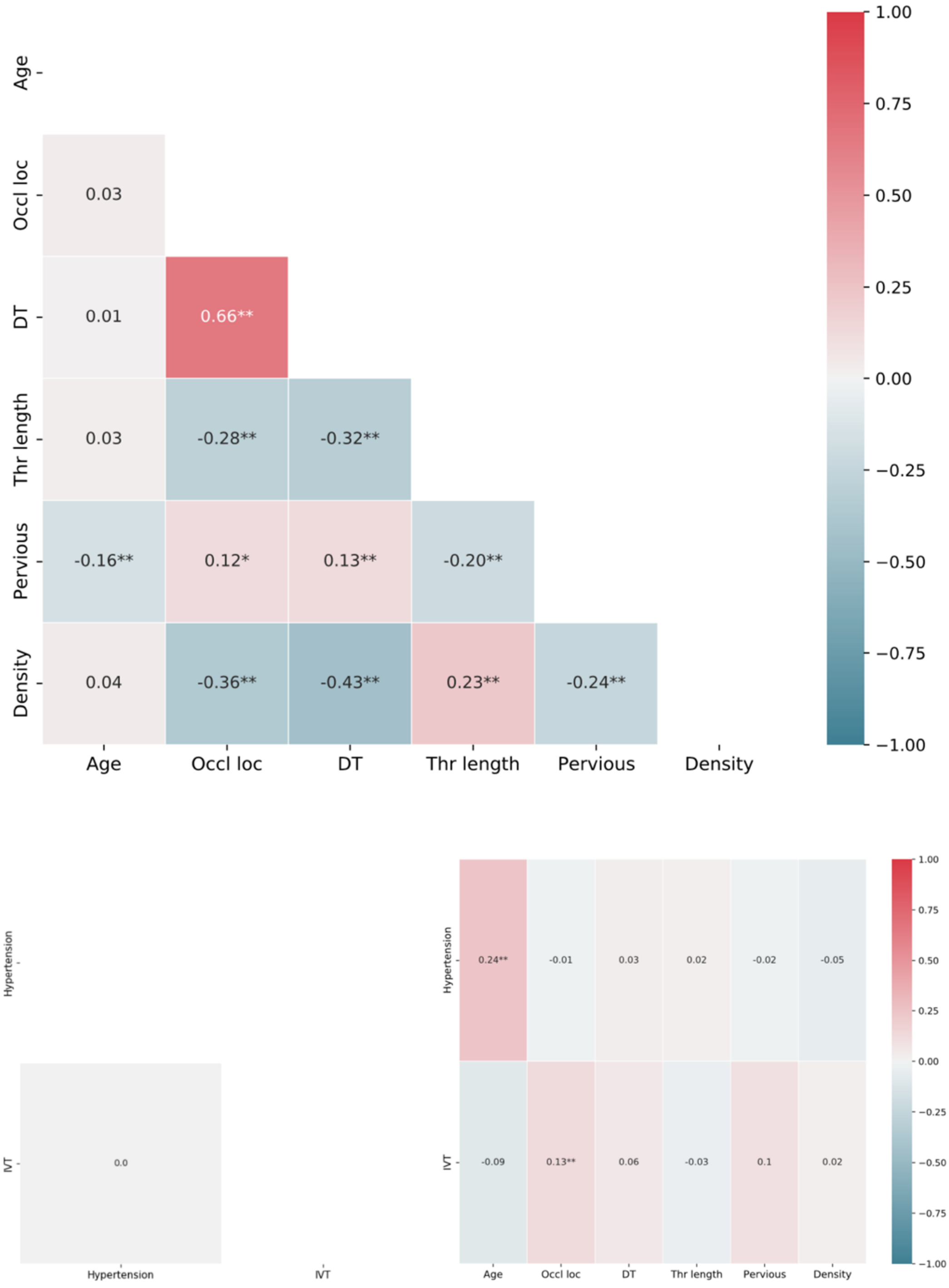

Supplemental Figure S3. Correlation analysis. Top: Spearman's correlation coefficients and significance level. Bottom left: Cramer's V coefficients and significance level. Bottom right: Point-biserial correlation coefficients and significance level. '**', p<0.01; '*', p<0.05; ' ' p≥0.05. Numerical variables: age, DT, thrombus length, thrombus perviousness and thrombus density. Categorical ordinal variables: occlusion location. Categorical binary variables: history of hypertension and IVT administration. *DT, distance from the intracranial carotid artery terminus to the thrombus; IVT, intravenous treatment with alteplase; loc, location; occl, occlusion; pervious, perviousness; thr, thrombus.*

MR CLEAN Registry investigators – Group author

Executive committee
Diederik W.J. Dippel[1]; Aad van der Lugt[2]; Charles B.L.M. Majoie[3]; Yvo B.W.E.M. Roos[4]; Robert J. van Oostenbrugge[5]; Wim H. van Zwam[6]; Jelis Boiten[14]; Jan Albert Vos[8].

Study coordinators
Ivo G.H. Jansen[3]; Maxim J.H.L. Mulder[1,2]; Robert- Jan B. Goldhoorn[5,6]; Kars C.J. Compagne[2]; Manon Kappelhof[3]; Josje Brouwer[4]; Sanne J. den Hartog[1,2,40]; Wouter H. Hinsenveld [5,6].

Local principal investigators
Diederik W.J. Dippel[1]; Bob Roozenbeek[1]; Aad van der Lugt[2]; Adriaan C.G.M. van Es[2]; Charles B.L.M. Majoie[3]; Yvo B.W.E.M. Roos[4]; Bart J. Emmer[3]; Jonathan M. Coutinho[4]; Wouter J. Schonewille[7]; Jan Albert Vos[8]; Marieke J.H. Wermer[9]; Marianne A.A. van Walderveen[10]; Julie Staals[5]; Robert J. van Oostenbrugge[5]; Wim H. van Zwam[6]; Jeannette Hofmeijer[11]; Jasper M. Martens[12]; Geert J. Lycklama à Nijeholt[13]; Jelis Boiten[14]; Sebastiaan F. de Bruijn[15]; Lukas C. van Dijk[16]; H. Bart van der Worp[17]; Rob H. Lo[18]; Ewoud J. van Dijk[19]; Hieronymus D. Boogaarts[20]; J. de Vries[22]; Paul L.M. de Kort[21]; Julia van Tuijl[21]; Jo P. Peluso[26]; Puck Fransen[22]; Jan S.P. van den Berg[22]; Boudewijn A.A.M. van Hasselt[23]; Leo A.M. Aerden[24]; René J. Dallinga[25]; Maarten Uyttenboogaart[28]; Omid Eschgi[29]; Reinoud P.H. Bokkers[29]; Tobien H.C.M.L. Schreuder[30]; Roel J.J. Heijboer[31]; Koos Keizer[32]; Lonneke S.F. Yo[33]; Heleen M. den Hertog[22]; Tomas Bulut[35]; Paul J.A.M. Brouwers[34].

Imaging assessment committee
Charles B.L.M. Majoie[3] (chair); Wim H. van Zwam[6]; Aad van der Lugt[2]; Geert J. Lycklama à Nijeholt[13]; Marianne A.A. van Walderveen[10]; Marieke E.S. Sprengers[3]; Sjoerd F.M. Jenniskens[27]; René van den Berg[3]; Albert J. Yoo[38]; Ludo F.M. Beenen[3]; Alida A. Postma[6]; Stefan D. Roosendaal[3]; Bas F.W. van der Kallen[13]; Ido R. van den Wijngaard[13]; Adriaan C.G.M. van Es[2]; Bart J. Emmer[3]; Jasper M. Martens[12]; Lonneke S.F. Yo[33]; Jan Albert Vos[8]; Joost Bot[36]; Pieter-Jan van Doormaal[2]; Anton Meijer[27]; Elyas Ghariq[13]; Reinoud P.H. Bokkers[29]; Marc P. van Proosdij[37]; G. Menno Krietemeijer[33]; Jo P. Peluso[26]; Hieronymus D. Boogaarts[20]; Rob Lo[18]; Wouter Dinkelaar[2]; Auke P.A. Appelman[29]; Bas Hammer[16]; Sjoert Pegge[27]; Anouk van der Hoorn[29]; Saman Vinke[20].

Writing committee
Diederik W.J. Dippel[1] (chair); Aad van der Lugt[2]; Charles B.L.M. Majoie[3]; Yvo B.W.E.M. Roos[4]; Robert J. van Oostenbrugge[5]; Wim H. van Zwam[6]; Geert J. Lycklama à Nijeholt[13]; Jelis Boiten[14]; Jan Albert Vos[8]; Wouter J. Schonewille[7]; Jeannette Hofmeijer[11]; Jasper M. Martens[12]; H. Bart van der Worp[17]; Rob H. Lo[18].

Adverse event committee
Robert J. van Oostenbrugge[5] (chair); Jeannette Hofmeijer[11]; H. Zwenneke Flach[23].

Trial methodologist
Hester F. Lingsma[40].

Research nurses / local trial coordinators
Naziha el Ghannouti[1]; Martin Sterrenberg[1]; Wilma Pellikaan[7]; Rita Sprengers[4]; Marjan Elfrink[11]; Michelle Simons[11]; Marjolein Vossers[12]; Joke de Meris[14]; Tamara Vermeulen[14]; Annet Geerlings[19]; Gina van Vemde[22]; Tiny Simons[30]; Gert Messchendorp[28]; Nynke Nicolaij[28]; Hester Bongenaar[32]; Karin Bodde[24]; Sandra Kleijn[34]; Jasmijn Lodico[34]; Hanneke Droste[34]; Maureen Wollaert[5]; Sabrina Verheesen[5]; D. Jeurrissen[5]; Erna Bos[9]; Yvonne Drabbe[15]; Michelle Sandiman[15]; Nicoline Aaldering[11]; Berber Zweedijk[17]; Jocova Vervoort[21]; Eva Ponjee[22]; Sharon Romviel[19]; Karin Kanselaar[19]; Denn Barning[10].

PhD / Medical students:
Esmee Venema[40]; Vicky Chalos[1,40]; Ralph R. Geuskens[3]; Tim van Straaten[19]; Saliha Ergezen[1]; Roger R.M. Harmsma[1]; Daan Muijres[1]; Anouk de Jong[1]; Olvert A. Berkhemer[1,3,6]; Anna M.M. Boers[3,39]; J. Huguet[3]; P.F.C. Groot[3]; Marieke A. Mens[3]; Katinka R. van Kranendonk[3]; Kilian M. Treurniet[3]; Manon L. Tolhuisen[3,39]; Heitor Alves[3]; Annick J. Weterings[3], Eleonora L.F. Kirkels[3], Eva J.H.F. Voogd[11]; Lieve M. Schupp[3]; Sabine L. Collette[28,29]; Adrien E.D. Groot[4]; Natalie E. LeCouffe[4]; Praneeta R. Konduri[39]; Haryadi Prasetya[39]; Nerea Arrarte-Terreros[39]; Lucas A. Ramos[39].

List of affiliations
Department of Neurology[1], Radiology[2], Public Health[40], Erasmus MC University Medical Center;
Department of Radiology and Nuclear Medicine[3], Neurology[4], Biomedical Engineering & Physics[39], Amsterdam UMC, University of Amsterdam, Amsterdam;
Department of Neurology[5], Radiology[6], Maastricht University Medical Center and Cardiovascular Research Institute Maastricht (CARIM);
Department of Neurology[7], Radiology[8], Sint Antonius Hospital, Nieuwegein;
Department of Neurology[9], Radiology[10], Leiden University Medical Center;
Department of Neurology[11], Radiology[12], Rijnstate Hospital, Arnhem;
Department of Radiology[13], Neurology[14], Haaglanden MC, the Hague;
Department of Neurology[15], Radiology[16], HAGA Hospital, the Hague;
Department of Neurology[17], Radiology[18], University Medical Center Utrecht;
Department of Neurology[19], Neurosurgery[20], Radiology[27], Radboud University Medical Center, Nijmegen;
Department of Neurology[21], Radiology[26], Elisabeth-TweeSteden ziekenhuis, Tilburg;
Department of Neurology[22], Radiology[23], Isala Klinieken, Zwolle;
Department of Neurology[24], Radiology[25], Reinier de Graaf Gasthuis, Delft;
Department of Neurology[28], Radiology[29], University Medical Center Groningen;
Department of Neurology[30], Radiology[31], Atrium Medical Center, Heerlen;
Department of Neurology[32], Radiology[33], Catharina Hospital, Eindhoven;
Department of Neurology[34], Radiology[35], Medisch Spectrum Twente, Enschede;
Department of Radiology[36], Amsterdam UMC, Vrije Universiteit van Amsterdam, Amsterdam;
Department of Radiology[37], Noordwest Ziekenhuisgroep, Alkmaar;
Department of Radiology[38], Texas Stroke Institute, Texas, United States of America.
